# Prediction of axillary lymph node pathological complete response to neoadjuvant therapy using nomogram and machine learning methods

**DOI:** 10.3389/fonc.2022.1046039

**Published:** 2022-10-24

**Authors:** Tianyang Zhou, Mengting Yang, Mijia Wang, Linlin Han, Hong Chen, Nan Wu, Shan Wang, Xinyi Wang, Yuting Zhang, Di Cui, Feng Jin, Pan Qin, Jia Wang

**Affiliations:** ^1^ Department of Breast Surgery, The Second Hospital of Dalian Medical University, Dalian, China; ^2^ Faculty of Electronic Information and Electrical Engineering, Dalian University of Technology, Dalian, China; ^3^ Health Management Center, The Second Hospital of Dalian Medical University, Dalian, China; ^4^ Information Center, The Second Hospital of Dalian Medical University, Dalian, China; ^5^ Department of Breast Surgery, The First Affiliated Hospital of China Medical University, Shenyang, China

**Keywords:** breast cancer, axillary lymph node pathological complete response, neoadjuvant therapy, nomogram, machine learning

## Abstract

**Purpose:**

To determine the feasibility of predicting the rate of an axillary lymph node pathological complete response (apCR) using nomogram and machine learning methods.

**Methods:**

A total of 247 patients with early breast cancer (eBC), who underwent neoadjuvant therapy (NAT) were included retrospectively. We compared pre- and post-NAT ultrasound information and calculated the maximum diameter change of the primary lesion (MDCPL): [(pre-NAT maximum diameter of primary lesion – post-NAT maximum diameter of preoperative primary lesion)/pre-NAT maximum diameter of primary lesion] and described the lymph node score (LNS) (1): unclear border (2), irregular morphology (3), absence of hilum (4), visible vascularity (5), cortical thickness, and (6) aspect ratio <2. Each description counted as 1 point. Logistic regression analyses were used to assess apCR independent predictors to create nomogram. The area under the curve (AUC) of the receiver operating characteristic curve as well as calibration curves were employed to assess the nomogram’s performance. In machine learning, data were trained and validated by random forest (RF) following Pycharm software and five-fold cross-validation analysis.

**Results:**

The mean age of enrolled patients was 50.4 ± 10.2 years. MDCPL (odds ratio [OR], 1.013; 95% confidence interval [CI], 1.002–1.024; *p*=0.018), LNS changes (pre-NAT LNS – post-NAT LNS; OR, 2.790; 95% CI, 1.190–6.544; *p*=0.018), N stage (OR, 0.496; 95% CI, 0.269–0.915; *p*=0.025), and HER2 status (OR, 2.244; 95% CI, 1.147–4.392; *p*=0.018) were independent predictors of apCR. The AUCs of the nomogram were 0.74 (95% CI, 0.68–0.81) and 0.76 (95% CI, 0.63–0.90) for training and validation sets, respectively. In RF model, the maximum diameter of the primary lesion, axillary lymph node, and LNS in each cycle, estrogen receptor status, progesterone receptor status, HER2, Ki67, and T and N stages were included in the training set. The final validation set had an AUC value of 0.85 (95% CI, 0.74–0.87).

**Conclusion:**

Both nomogram and machine learning methods can predict apCR well. Nomogram is simple and practical, and shows high operability. Machine learning makes better use of a patient’s clinicopathological information. These prediction models can assist surgeons in deciding on a reasonable strategy for axillary surgery.

## Introduction

Neoadjuvant therapy (NAT) is a systemic treatment that precedes local surgery and not only monitors the response to systemic therapy, but also offers patients with early breast cancer (eBC) a higher rate of breast-conserving surgery and omission from axillary lymph node dissection (ALND) (1). While traditional anatomic pathological features are important in predicting the risk of recurrence and deciding on adjuvant treatment options for patients with eBC, the response of the primary breast lesion and lymph node post-NAT are also important for any subsequent adjuvant treatment regimen (2). At the same time, when patients achieve an axillary lymph node pathological complete response (apCR) post-NAT, an opportunity exists to omit ALND and avoid postoperative complications such as lymphedema, arm pain, and arm dyskinesia (3). According to the results of Z1071 (4), SENTINA (5), and SN FNAC (6) trials, although the proportion of patients who achieved apCR post-NAT was within acceptable range (40%–70%), the proportion of patients with a negative clinical evaluation of axillary lymph node post-NAT who obtained an ALND exemption through sentinel lymph node biopsy (SLNB) was small in the real world. Although the National Cancer Database shows that 42.2% of NAT patients are exempted from ALND by SLNB (7), many countries and regions show a far lower ALND rate. A cross-sectional survey of 110 large hospitals in mainland China showed that more than 50% of hospitals preferred to perform SLNB before NAT. If SLNB is positive at this time point, further ALND is required and the opportunity to perform SLNB post-NAT is lost (8). The reasons for this may be because of the high false negative rate (FNR) of 8.4–14.2% for SLNB. Although a subgroup analysis of the Z1071 trial found that the placement of marker clips in positive lymph nodes reduced the FNR of post-NAT sentinel lymph node biopsies to 6.8%, 20% of marker clips were not placed in sentinel lymph nodes and 17% were lost (9). If SLNB was only performed for patients with no suspicious lymph nodes on a post-NAT ultrasound, the FNR decreased to 9.8%. But of 138 patients with suspicious lymph nodes on ultrasound, no metastases were found in the postoperative lymph node pathology of 39 patients (10). However, the poor implementation of radioisotope and placement of marker clips that occurred in many medical institutions also influenced the performance of post-NAT SLNB. Therefore, the development of other practical tools is urgently required to screen for an appropriate population for post-NAT SLNB.

Previous studies have described such a prediction model, which were based on pre-and post-NAT clinicopathological and imaging information (11–14). One study included data on suspicious lymph nodes on post-NAT ultrasound in the prediction model to improve accuracy (15). Unfortunately, the dynamic changes in lymph node status post-NAT were not considered. A meta-analysis showed that the accuracy of ultrasound evaluation for lymph node status post-NAT was only 0.58 (16). In addition, machine learning to predict total pCR (tpCR) and long-term survival in patients with NAT eBC has been developed in several studies. A study based on machine learning to predict tpCR in patients with eBC had the highest AUC value of 0.87 but required additional genomic and transcriptomic profiles from patients (17). A report using multiparameter magnetic resonance imaging (MRI) combined with machine learning to predict tpCR and survival had the highest AUC value of 0.86; only imaging features of the primary lesion were extracted and the characteristics of lymph nodes were ignored (18). Meti et al. combined tumor size, histological grade, clinical stage, and molecular subtype to construct a prediction model, but did not include imaging features (19). Thus, these latest studies suggested an urgent need exists for developing a tool that can accurately predict the status of axillary lymph node post-NAT through routine examination and clinical information.

Therefore, the aim of our study was to predict the post-NAT lymph node status of patients with eBC using clinicopathological and ultrasound information during NAT by nomogram and random forest (RF) methods respectively, and to compare the advantages and disadvantages of the two methods. We expect that these models may provide clinicians with a simple, accurate, and easy-to-use method for predicting a candidate population for SLNB post-NAT, thus avoiding complex technical approaches.

## Methods

### Study population

Data on a total of 382 patients with eBC, who underwent NAT at the Second Hospital of Dalian Medical University and the First Affiliated Hospital of China Medical University between 2017 and 2021, were collected retrospectively. The eligibility criteria for the study were as follows (1): at least four cycles of NAT (2); surgery post-NAT (3); complete ultrasound examination in all treatment cycles (4); complete pathological information (5); treatment regimen containing anthracyclines and/or paclitaxel; and (6) a primary lesion detected on breast ultrasound.

**Figure 1:**
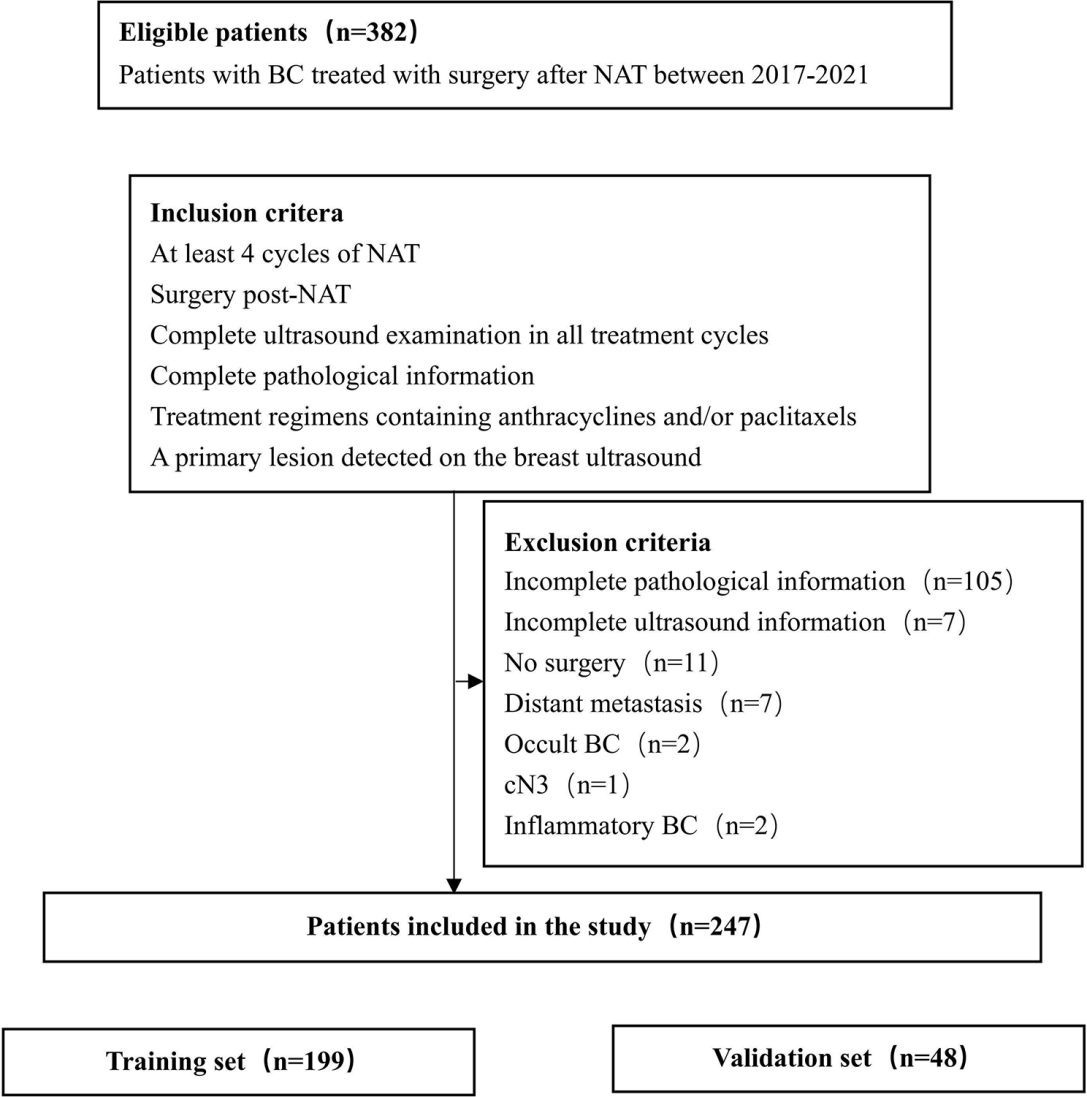
Study flowchart.

Based on the medical history and imaging findings, we excluded (1): incomplete pathological information (2); incomplete ultrasound information (3); no surgery (4); distant metastasis (5); occult breast cancer (BC) (6); cN3; and (7) inflammatory BC (**Figure 1**). In total, 247 patients were included in the study.

### Data collection

We collected clinical information including (1): age, and (2) pre-NAT clinical T and N stages. Pre- and post-NAT pathological information included (1): estrogen receptor (ER) status (2); progesterone receptor (PR) status (3); human epidermal growth factor receptor 2 (HER2) status; and (4) Ki-67 index. TNM staging was performed according to the 8th edition of the American Joint Committee on Cancer Staging Manual (20). ER and PR expression ≥ 1% was considered positive (21). ER or PR positive was considered hormone receptor (HR) positive (22). Immunohistochemistry (IHC) 3+ was considered HER2 positive. IHC 2+ was tested using fluorescence *in situ* hybridization (FISH): a HER2/CEP17 ratio ≥ 2.0 with a HER2 signal/cell ratio ≥ 4.0 or a HER2/CEP17 ratio < 2.0 with a HER2 signal/cell ≥ 6.0 were positive (23). Breast pCR (bpCR) was defined as no invasive disease in the breast and apCR was defined as no metastatic disease in an axillary lymph node (24) including isolated tumor cells and micrometastases (25). Total pCR (tpCR) was defined as ypTis/0N0M0.

### Ultrasound

**Figure 2:**
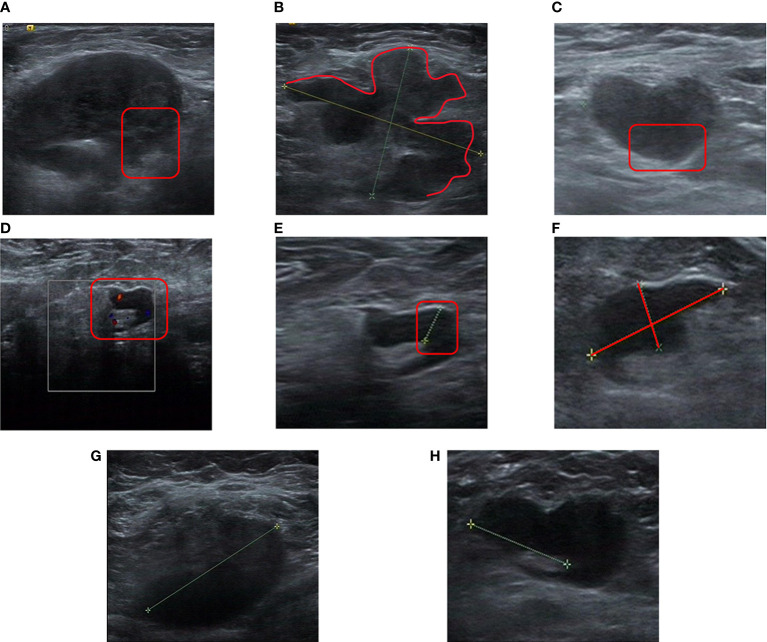
Specific image features for a lymph node score. **(A)**, Unclear border (As shown in the red box, the border of the lymph node is unclear from the surrounding tissue). **(B)**, Irregular morphology (as shown in the red line, the loss of an elliptical shape led to a lobulated shape). **(C)**, Absence of hilum (the lymph nodes shown in the red boxes were almost all hypoechoic, and no echo of the hilum was seen). **(D)**, Visible vascularity (vascularity is visible in the cortex and hilum shown in the box). **(E)**, Cortical thickness (significant cortical thickness shown in the red box). **(F)**, Aspect ratio <2 (ratio of vertical axis to horizontal axis <2). **(G)**, Unclear border, irregular morphology, absence of hilum, cortical thickness (LNS = 4 points). **(H)**, Unclear border, irregular morphology, cortical thickness (LNS = 3 points). LNS, lymph node score.

All patients ultimately included in the study underwent breast ultrasound during each cycle of NAT. The maximum diameter of the primary lesion (MDPL) and maximum diameter of a suspicious lymph node (MDSLN) were recorded for each cycle. The maximum diameter change of the primary lesion (MDCPL) was calculated: [(pre-NAT MDPL – post-NAT MDPL)/pre-NAT MDPL]. If multiple lesions were detected, information on the lesion with the largest diameter was selected. An ultrasound evaluation was made of the following suspicious axillary lymph node features (1): unclear border (**Figure 2A**) (2); irregular morphology (**Figure 2B**) (3); absence of hilum (**Figure 2C**) (4); visible vascularity (**Figure 2D**) (5); cortical thickness (**Figure 2E**); and (6) aspect ratio <2 (**Figure 2F**) (26–29). The lymph node score (LNS) was set up, with each abnormal description considered as a score of 1. For example, a NAT patient with lymph node ultrasound images showing an unclear border, irregular morphology, absence of hilum, and cortical thickness would have an LNS of 4 points (**Figure 2G**). Another patient with lymph node ultrasound images showing an unclearborder, irregular morphology and cortical thickness would have an LNSof 3 points (**Figure 2H**). The difference between pre- and post-NAT was calculated and divided into two groups, with 0 as the cutoff value (1): ≥0 (2) <0.

### Machine learning

Machine learning was randomly divided into training and validation sets and stratified 7:3. The feature values of the training set sample data included patient pre-NAT T stage, N stage, MDPL and MDSLN for each cycle, LNS for each cycle, ER expression, PR expression, Ki67, and HER2 status.

The RF method is an integrated classification method (30). This model is a commonly used classifier in the intersectional field of medicine and artificial intelligence. It is composed of multiple decision trees and the output category is determined by majority voting according to the output category of each tree.

The software environment used in this study was Pycharm and the Python interpreter version was 3.8.12. First, the training and validation sets were stratified and randomly divided 7:3 to ensure a consistent proportion of apCR and non-apCR sample data. Second, the feature values of the sample data were standardized to eliminate differences between features. In view of the imbalance in the number of samples of various types in the data set, SMOTE synthesis method was used to oversampling the minority sample data (patients who reached apCR), so as to improve the recognition ability of the model for minority samples. When training the model, we try to use different class weight to adjust the influence degree of different types of training data on the loss function, in order to achieve the best classification effect. With the aim of the problem being that the total sample data was small, in the process of selecting the optimal parameters for the training model, this experiment used a cross-validation method to compare the advantages and disadvantages of the model with different parameters in the prediction result index on the validation set. Finally, after determining the parameters, all sample data of the training set was inputted to train the model, the final prediction results of the validation set on the model were saved, the confusion matrix and receiver operating characteristic (ROC) curve were drawn, and the performance index accuracy, sensitivity, specificity and area under the curve (AUC) of the ROC curve were calculated. For this experimental dataset, which was classified with unbalanced labels, the error could be well balanced. Python language was used in its programming part. In order to obtain a stable and reliable model, this experiment adopted a 5-fold cross-validation method to select parameters. Finally, the ROC was drawn based on predicted and true label values on the model. The AUC of the ROC curve was calculated as the performance index of the model.

### Statistical analysis

Randomization into training and validation sets was performed at 8:2. We used logistic regression to obtain an odds ratio (OR) with 95% confidence interval (CI) for any association with the response. Univariable logistic regression was used to explore the clinicopathological and ultrasound factors associated with lymph node pCR. Variables with *p* < 0.05 in the univariable logistic regression were included in the multivariable logistic regression. Variables with *p* < 0.05 in the multivariable logistic regression were retained in the nomogram. The AUCs of the ROC and calibration curves were employed to assess the nomogram’s performance (31). Statistical analyses were carried out in SPSS 25 and R (version 4.2.1) software. In addition, “glm,” “rms,” “pROC,” “Calibration Curves” packages were used.

## Results

### Pre-NAT characteristics

In total, 247 patients were included in this retrospective study. The pre-NAT clinical and pathological characteristics of patients in the training and validation sets are shown in **Table 1**. The mean patient age ± standard deviation was 50.4 ± 10.2 years. The majority of patients with a T stage were T2 at 64% (158 of 247) of the population, followed by T3–T4 patients accounting for 27.5% (68 of 247), and T1 patients accounting for a minimum of 8.5% of the population (21 of 247). The highest percentage of patients with an N1 stage was 73.7% (182 of 247). 97.6% of patients were invasive ductal carcinoma (IDC). We classified molecular subtypes into (1): HR+/HER2- (2), HR+/HER2+ (3), HR-/HER2-, and (4) HR-/HER2+. The most frequent were HR+/HER2- at 49.8% (123 out of 247) of patients, and HR+/HER2+ at 23.5% (58 of 247) of patients. The rest of the types were HR-/HER2- at 12.5% (31 of 247) of patients and HR-/HER2+ at 14.2% (35 of 247) of patients, respectively. Anti-HER2 drugs were used in 81% (75 of 93) of HER2+ patients. The tpCR rate was 23.9%, and the rates for bpCR and apCR were 26.7% and 48.1%, respectively (**Table 2**).

**Table 1:** Basic characteristics of patients at baseline.

Characteristic	All	Training set	Validation set
	n=247	n=199	n=48
Age	50.3 ± 10.2	50.5 ± 10.2	49.6 ± 10.8
T stage			
1	21(8.5%)	17(8.5%)	4(8.3%)
2	158(64%)	126(63.4%)	31(64.6%)
3、4	68(27.5%)	56(28.1%)	13(27.1%)
N stage			
0	33(13.3%)	26(13.1%)	7(14.5%)
1	182(73.7%)	147(73.9%)	35(73%)
2	32(13%)	26(13%)	6(12.5%)
Pathological type			
IDC^a^	241 (97.6%)	196 (98.5%)	45 (93.8%)
ILC^b^	2 (0.8%)	1 (0.5%)	1 (2.1%)
Other types	4 (1.6%)	2 (1%)	2 (4.1%)
Molecular subtype			
HR+/HER2-	123(49.8%)	95(47.7%)	28(58.2%)
HR+/HER2+	58(23.5%)	48(24.1%)	10(21%)
HR-/HER2-	31(12.5%)	24(12.1%)	7(14.6%)
HR-/HER2+	35(14.2%)	32(16.1%)	3(6.2%)
apCR rates	48.1%	48.7%	45.8%

a Invasive ductal carcinoma.

b Invasive lobular carcinoma.

**Table 2:** Breast, axillary lymph node and total pCR rates for different molecular subtypes.

Molecular subtype	bpCR rates	apCR rates	tpCR rates
All	26.7%	48.1%	23.9%
HR+/HER2-	17.9%	37.4%	16.3%
HR+/HER2+	32.8%	63.8%	31.0%
HR-/HER2-	19.4%	45.2%	16.1%
HR-/HER2+	54.3%	62.9%	45.7%

### Factors associated with apCR

In the training set (n=199), apCR was achieved in 48.7% of patients (**Table 1**). A total of 10 variables were included in the statistical analysis (**Table 3**). In a univariable logistic regression analysis, clinicopathological characteristics: ER expression (OR, 0.989; 95% CI, 0.982–0.996; *p* = 0.001), HER2 status (OR, 2.715; 95% CI, 1.511–4.876; *p* = 0.001), Ki67 index (OR, 6.560. 95% CI, 1.457–29.532; *p* = 0.014), and N stage (OR, 0.554; 95% CI, 0.319–0.962; *p* = 0.036) were associated with apCR. For ultrasound features, MDCPL (OR, 1.015; 95% CI, 1.005–1.025; *p* = 0.004) and LNS changes (OR, 3.650; 95% CI, 1.621–8.220; *p* = 0.002) were associated with apCR. The p values for T stage, PR expression, molecular subtype and age were less than 0.05 so they were not included in the multivariable logistic regression analysis (**Table 3**).

**Table 3:** Results of variables associated with apCR in univariable logistic regression analysis.

Characteristic	P value	OR	95%CI
MDCPL	0.004	1.015	1.005-1.025
LNS changes	0.002	3.650	1.621-8.220
T stage	0.619	0.884	0.544-1.437
N stage	0.036	0.554	0.319-0.962
ER expression	0.001	0.989	0.982-0.996
PR expression	0.133	0.501	0.204-1.235
HER2 status	0.001	2.715	1.511-4.876
Ki67 index	0.014	6.560	1.457-29.532
Molecular subtype	0.371	1.326	0.714-2.462
Age	0.833	0.997	0.970-1.025

The above variables with *p* < 0.05 were included in a multivariable logistic regression analysis, which showed that the MDCPL (OR, 1.013; 95% CI, 1.002–1.024; *p* = 0.018), LNS changes (OR, 2.790; 95% CI, 1.190–6.544; *p* = 0.018), N stage (OR, 0.496; 95% CI, 0.269–0.915; *p* = 0.025), and HER2 status (OR, 2.244; 95% CI, 1.147–4.392; *p* = 0.018) were associated with apCR (**Table 4**).

**Table 4:** Results of variables associated with apCR in multivariable logistic regression analysis.

Characteristic	P value	OR	95%CI
MDCPL	0.018	1.013	1.002-1.024
LNS changes	0.018	2.790	1.190-6.544
N stage	0.025	0.496	0.269-0.915
HER2 status	0.018	2.244	1.147-4.392
ER expression	0.104	0.993	0.985-1.001

### Nomogram construction and validation

**Figure 3:**
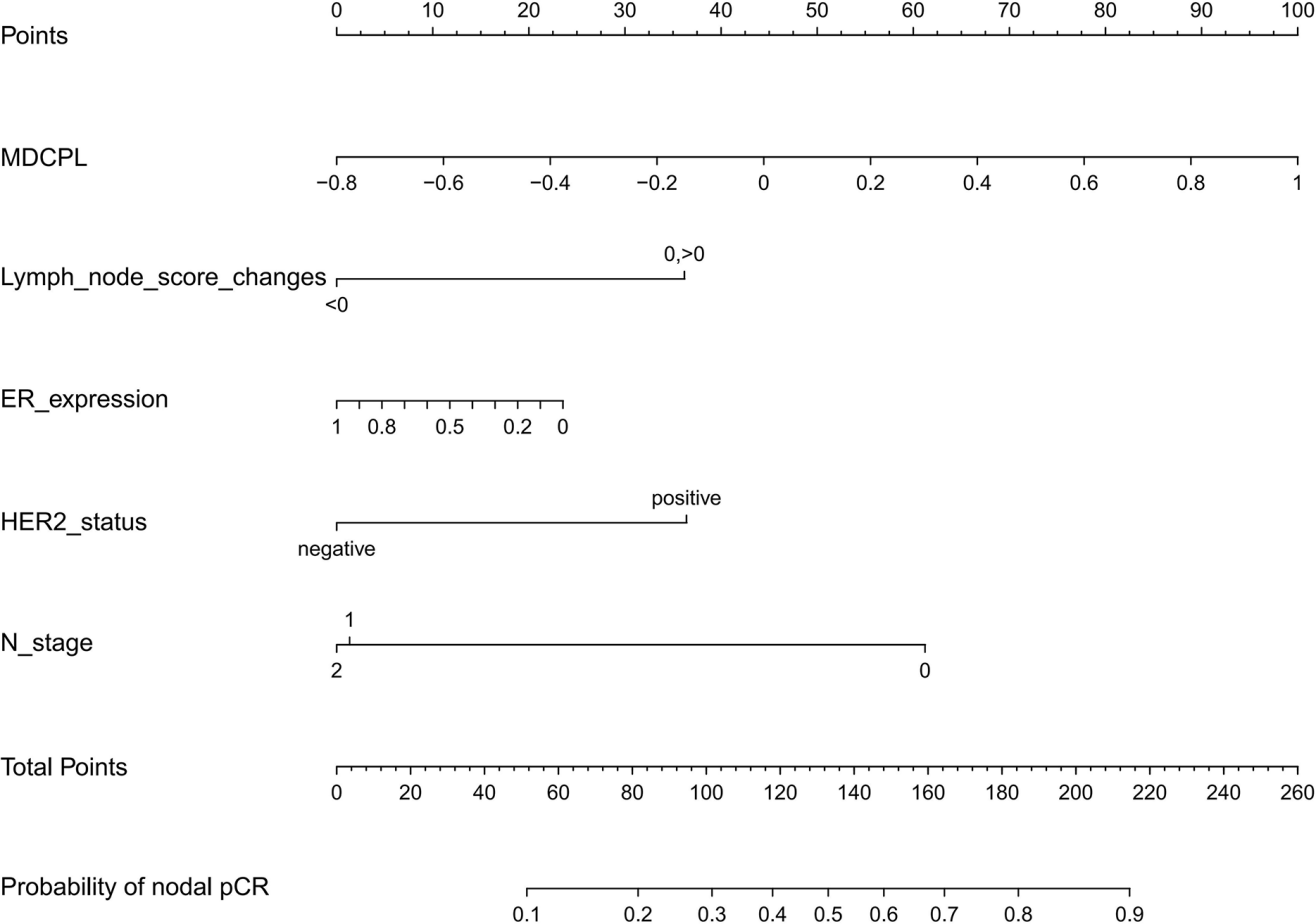
Nomogram for predicting the probability of apCR. Variables including MDCPL, LNS changes, percentage ER expression, HER2 status, and N stage were assigned points. Adding the total score of these variable points indicates the probability of apCR. The vertical line between the five variables and the first row can be summed as a total point, and by drawing a vertical line between the total point and the last row, the final probability of apCR was obtained. apCR, axillary lymph node pathological complete response; ER, estrogen receptor; HER2, human epidermal growth factor receptor 2; LNS, lymph node score; MDCPL, maximum diameter change of primary lesion.

The ER was a predictor of apCR in previous studies (31–34). Although we did not see a statistically significant difference in ER expression in a multivariable logistic regression analysis (OR, 0.993; 95% CI, 0.985–1.001; *p* = 0.104), we still included ER expression in the nomogram. The total score of the nomogram was obtained by summing the respective scores of MDCPL, ER expression, LNS changes, HER2 status, and N stage. The probability of achieving apCR for an individual patient was obtained by applying the total score to the scale at the bottom of the nomogram (**Figure 3**).

**Figure 4:**
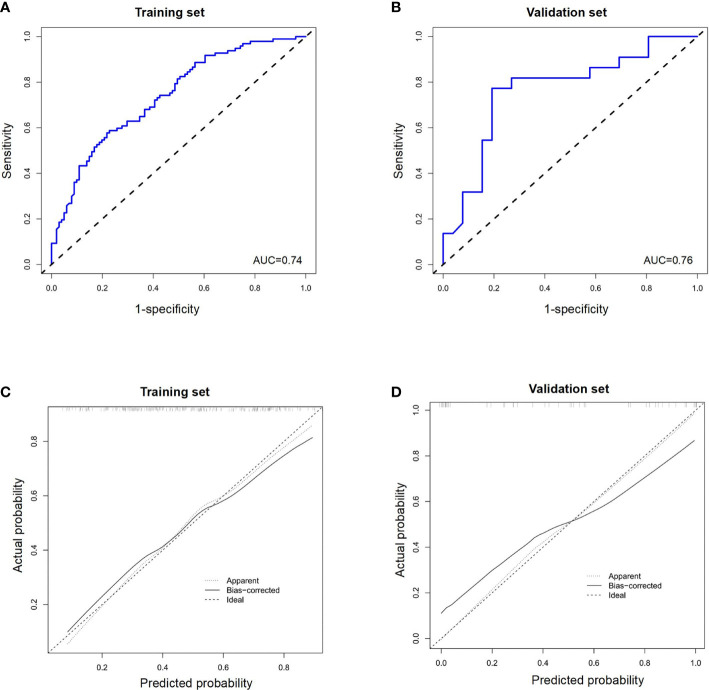
The receiver operating characteristic (ROC) curves of a nomogram for predicting the probability of apCR in **(A)** the training set and **(B)** validation set. Calibration curves of a nomogram for predicting the probability of apCR in the training set **(C)** and validation set **(D)**. apCR, axillary lymph node pathological complete response.

Internal validation on a training set with a AUC value of 0.74 (95% CI, 0.68–0.81) and independent external validation on a validation set with a AUC value of 0.76 (95% CI, 0.63–0.90) indicated that the reformulated model had good predictive ability (**Figures 4A, B**). The calibration curve of the nomogram showed good agreement between actual observations and predicted outcomes in training and validation sets (**Figures 4C, D**). As the prediction curves were close to the standard curve (Y=X), the final model showed good performance and had high practicability.

### Validation of machine learning (RF model construction)

**Figure 5:**
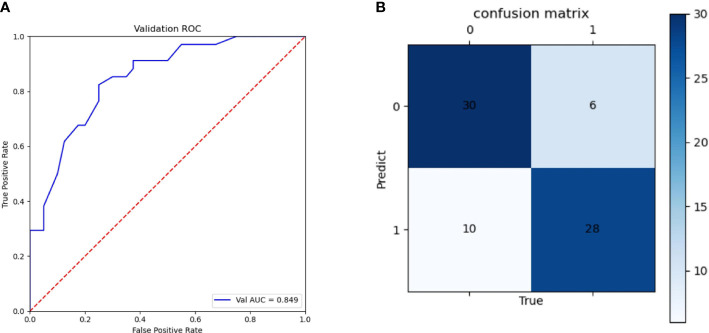
The receiver operating characteristic (ROC) curve of a random forest (RF) model in the validation set **(A)** and confusion matrix of a random forest (RF) model **(B)**.

In this study, we used features that were already available in the data; however, the values of individual features needed to be preprocessed. First, the values of ER, PR, and HER2 were binarized according to the critical values of negative and positive in medicine, and the remaining eigenvalues were retained. The processed training set data were sent to the RF model for training, and the appropriate model parameters were selected by comparing the mean of the 5-fold cross-validation result. Finally, pre-NAT T stage, N stage, MDPL and MDSLN for each cycle, LNS for each cycle, ER expression, PR expression, Ki67, and HER2 status were included in the model and the performance of the model was examined in the validation set. The indicators used in this experiment were AUC, accuracy, sensitivity, specificity, and ROC (**Figure 5A**), showing an AUC value of 0.85 (95% CI, 0.74–0.87). The confusion matrix in **Figure 5B** shows that the RF model has a prediction accuracy of 0.78, sensitivity of 0.74, and specificity of 0.83. These results indicated that this model was predictive.

## Discussion

Due to the iterative update of new targeted drugs and the increasing refinement of molecular subtype, the proportion of patients with eBC who achieve surgical downstaging through NAT is increasing (34). Thus, more patients have conditions that lead to preservation of breast and axillary lymph nodes, especially those with triple-negative and HER2+ BC (35). Previous studies have shown better disease free survival (DFS) and overall survival (OS) rates could be obtained in patients who achieved pCR compared to those with residual lesions (22). An OS benefit is also seen in some subtypes with pCR in a single breast or axilla (36, 37). Also, several studies have reported that axillary lymph nodes appear to be more likely to reach pCR compared to the breast (2, 38, 39). A total of 48.1% of patients in our study achieved apCR, similar to previous studies (40–42). It is essential to perform an SLNB for patients who may then be exempted from ALND after NAT. How to select such feasible SLNB candidates by scientific methods is the key to constraining this problem.

The 2022 edition of the National Comprehensive Cancer Network breast cancer guidelines (43), and the 2021 edition of the Chinese breast cancer guidelines (CBCS, CSCO) (44, 45), generally recommend SLNB in selected cases when nodes are clinically negative after NAT. Conditions for SLNB were the pre-NAT placement of marker clips and their removal during surgery, use of dual tracers including radioisotopes, and removal of at least three sentinel lymph nodes. The rates for the utilization of radioisotope tracing in Z1071 (4), SENTINA (5), and SNFNAC (6) trials were 95.9%, 95%, and 100%, respectively. Unfortunately, the clinical prevalence of radioisotopes and marker clips has limited the widespread implementation of SLNB after NAT in many countries such as China. A cross-sectional study of 110 large hospitals in mainland China showed that only 14.5% of hospitals chose dual tracers containing a radioisotope (46). Another cross-sectional study showed that only 11% of hospitals chose to localize primary breast lesions and lymph nodes with marker clips (8).

Our study was designed to address this clinical challenge by using a scientific approach to help clinicians screen for patients who can truly avoid ALND through monitoring the axillary lymph node response of NAT dynamically. We also sought to develop an effective method for SLNB post-NAT that does need not be restricted by conditions, such as radioisotopes and marker clips. In this study, a prediction model based on nomogram and RF methods was developed to discriminate patients who could undergo SLNB post-NAT. The AUC values of nomogram and RF methods were 0.76 and 0.85, respectively, and both methods showed good predictive ability. The variables incorporated in the final nomogram by logistic regression analysis were MDCPL, LNS changes, ER expression percentage, HER2 status, and pre-NAT N stage. The eigenvalues incorporated in the RF model were MDPL by cycle, MDSLN by cycle, LNS by cycle, T stage, N stage, ER, PR, HER2, and Ki67. In our study, whether nomogram or machine learning, the included variables can be obtained during routine examination, which does not require additional examination and is more consistent with actual clinical operation.

ER, HER2, and other IHC indicators have been shown to have predictive value for apCR post-NAT in several studies (11–14). Ultrasound is a general screening tool, with the advantage of being low-cost and can simultaneously assist in lymph node biopsies. A previous study shows that ultrasound is more accurate than mammography and MRI in the evaluation of axillary lymph nodes (47). Thus, the predictive value of ultrasound for axillary lymph nodes is controversial. The essential problem is the homogeneity of different medical centers cannot be guaranteed due to strong subjective judgment (sensitivity of 87% and specificity of 53%–97%) (48). Ultrasound features of metastatic lymph nodes are generally as follows (1): unclear border (2), irregular morphology (3), absence of hilum (4), visible vascularity (5), cortical thickness (6), and an aspect ratio <2 (26–29). It is usually thought that if one of these descriptions is met, the lymph node is likely to be a metastatic lymph node. However, our clinical experience has found that post-NAT ultrasound features showing metastatic lymph nodes postoperatively usually correlate with the number of these features present. A study found that the post-NAT ultrasound detection of suspicious lymph nodes predicted a lower rate of apCR (15). We believe that the various descriptions associated with abnormal lymph nodes may have inconsistent weights for evaluating lymph node metastasis. But we do not know whether they are the independent factors. Therefore, we originally established LNS as an observation index, assigning scores to each abnormal description, and calculating the difference pre- and post-NAT. Interestingly, patients with an LNS difference ≥0 are more likely to achieve apCR. To our knowledge, this is the first study to considerthe “LNS” as an independent factor influencing the evaluation of lymph node metastasis post-NAT.

NAT is a process, and therefore, patients’ primary breast and lymph node metastatic lesions also show a dynamic process of change as treatment advances. Whether such a change process is reflected in imaging features that can be suggestive in predicting pCR is also a recent hot topic. A study that predicted tpCR, by assessing the change in depth and width of primary foci described by ultrasound pre- and post-NAT, showed that the greater the reduction in depth of primary foci in triple-negative BC, the easier it was to achieve tpCR (49). Li et al. comparing ultrasound changes during NAT to predict apCR, found that the clinical response of the primary focus and the percentage of lymph nodes showing a reduction in their short diameter after NAT predicted apCR (50). The results of the above study suggest that changes in imaging features before and after NAT do have an important predictive value for pCR. This also inspired us to choose the pre-NAT and post-NAT LNS difference when conducting this study, instead of selecting the LNS at a separate time point pre- or post-NAT.

Moreover, the response of primary and metastatic axillary lymph nodes to NAT is generally consistent, but occasionally discordant cases occur, sometimes not exactly with the same treatment effect (51). We therefore ventured to see if the inclusion of MDCPL in the model would improve its predictive ability. In our study, an interesting phenomenon was observed was that some patients showed a trend of enlargement of the primary breast lesion while the axilla got pCR (6 of 27). Previous studies have shown that about 14.5% of patients show different post-NAT outcomes in breast and axillary lymph nodes (52). We therefore retained this group of patients and included them in the study.

The nomogram is simple to construct and easy to apply. The apCR probability can be derived by calculating the score from post-NAT clinical characteristics; however, some variables were excluded after statistical analysis that resulted in data compression. The RF model is more complex to construct than a nomogram, and its AUC results are greater than those of the nomogram. Machine learning can use all available clinicopathological information and prediction results are more reliable. However, when using this algorithm, the training time is relatively long. In addition, the process of data pre-processing and finding the optimal parameters of the model requires some knowledge of machine learning and practical experience in programming. This makes the whole process more tedious in general.

Our study has several limitations. First, this was a retrospective study with a limited number of enrolled patients, but efforts were made to collect data from different study centers in order to minimize sample selection bias. Second, it is generally accepted that MRI is more accurate than ultrasound for primary breast lesion assessment (53), but ultrasound was used for primary lesion assessment because of excessive missing MRI data. This is also in line with the Chinese context and we believe that it would be more in line with Chinese clinical practice if we could indeed find a way to use ultrasound to achieve increased accuracy in being predictive. Third, not all suspicious axillary lymph nodes by clinical evaluation pre-NAT in our study underwent biopsy, which may influence the results to a greater or lesser extent. We also plan to increase the numbers of observed samples and design a prospective study in future.

In conclusion, our study developed a practical prediction model to help clinicians decide on an optimal surgical approach for axillary lymph node post-NAT based on nomogram and RF algorithm methods. Both prediction models can lead to the accurate prediction of apCR and guide the mode of surgical intervention of axillary lymph nodes in order to avoid non-essential ALND and minimize injury.

## Data availability statement

The raw data supporting the conclusions of this article will be made available by the authors, without undue reservation.

## Author contributions

TZ conceived this study and wrote the manuscript. MY and PQ performed the machine learning part. MW assisted in writing the manuscript. LH and DC assisted in the evaluation of ultrasound. HC, NW, SW, YZ and XW assisted in the evaluation of ultrasound and revising the manuscript. FJ provided a part of data. JW designed the study and was the director for the fund. All authors read and approved the final manuscript.

## Funding

This study was supported by the “1+X” program cross-disciplinary innovation project (No. 2022JCXKYB07)

## Conflict of interest

The authors declare that the research was conducted in the absence of any commercial or financial relationships that could be construed as a potential conflict of interest.

## Publisher’s note

All claims expressed in this article are solely those of the authors and do not necessarily represent those of their affiliated organizations, or those of the publisher, the editors and the reviewers. Any product that may be evaluated in this article, or claim that may be made by its manufacturer, is not guaranteed or endorsed by the publisher.
